# ASO Author Reflections: From Radical Mastectomy to Extreme Oncoplasty in One Lifetime

**DOI:** 10.1245/s10434-026-20269-0

**Published:** 2026-07-21

**Authors:** Melvin J. Silverstein, Jenna Capuano

**Affiliations:** 1 Hoag Breast Center, Hoag Memorial Hospital Presbyterian, Newport Beach, CA USA; 2 Keck School of Medicine, University of Southern California, Los Angeles, CA USA

## Preface

The *Past* was written by MJS as he lived it. The *Present* was written by both of us. The *Future* was predicted by JC as she is about to live it. Both of us edited the entire manuscript.

## Past

I went to medical school and trained as a surgical resident during the 1960s, an incredibly transformative chaotic decade, probably the most important 10 years of my life. I was a medical student when John F. Kennedy and Malcolm X were assassinated. I was a resident when Robert F. Kennedy and Martin Luther King were assassinated. Civil rights workers were murdered. Children were bombed in the 16th Street Baptist church. The Civil Rights Act and the Voting Rights Act were passed. I listened to the “I have a Dream” speech. I watched riots at the 1968 Democratic Convention on television. I absorbed the turmoil surrounding me, all the time studying to be a surgeon and loving medicine.

When I could, I listened to music: an incredible decade—the Beatles, the Rolling Stones, Bob Dylan, Janis Joplin, Motown, the Beach Boys…and dozens more. I met people face to face, going to parties and gatherings and talking with them.

If I had time, I watched the CBS Evening News with Walter Cronkite. I trusted him. The Vietnam War was on every night. By the end of the war, 58,220 Americans with an average age of 23 had died along with 2–3 million Vietnamese. Americans were not proud of this war.

The doctor draft was left over from the Korean war and was up and running when I graduated medical school. Senior medical students generally volunteered (rather than be drafted) for military service through the Berry Plan, hoping to get a deferment that would allow completion of specialty training. I got lucky. I was deferred for 5 years and went into the Army as a fully trained surgeon.

The Women’s Movement started a revolution. There was Betty Friedan, Gloria Steinem, Shirley Chisholm, and thousands more, demanding what was rightfully theirs, equal pay, sexual freedom, and fixing the inequities of everyday life. That fight continues today.

The 1960s brought the space race. We sent men to the moon and brought them home safely. Americans were proud of that.

It was 10 years of incredible progress in some fields, but NOT for breast cancer. Breast cancer resided in the same neighborhood where it lived for the past 60 years with few changes in our ability to diagnose or treat the disease. There was no mammographic screening, no MRI, no PET scans. Those machines did not even exist. So, there was no early diagnosis. There were no borderline ADH/DCIS (atypical ductal hyperplastic/ductal carcinoma in situ) cases. We didn’t even know those terms, and there was no reconstruction. Breast implants had just been invented, and reconstruction after mastectomy was 20 years away from becoming an option. And, of course, there was no breast conservation. Even thinking about anything less than a mastectomy was heresy. There was just one treatment, the Halsted muscle-removing radical mastectomy. “*The Operation Women Feared Most*” according to Dr. William Nolan in “McCall’s Magazine,” a magazine you bought at the checkout line of the supermarket.

Surgery of the breast remained unchanged since 1900, except operating rooms were nicer, anesthesia was safer, a few antibiotics were available, but the experience was the same—*the one-stage procedure*. The patient went to the operating room not knowing if she had breast cancer. An open biopsy was done, then a frozen section. If benign, she left the OR with a band-aid, so to speak. If cancer was found, she was re-prepped, re-draped, and underwent an immediate radical mastectomy. There was no true informed consent, the way we think of that process today. When she woke up, if it was cancer, she was shocked, in tears, and deformed. Her life changed forever.

In the 1960s, breast cancer was not the nuanced heterogenous disease we know today. It was kind of simple, a single disease with a single treatment. The surgeon was *Captain of the Ship* and there was no such thing as the multidisciplinary approach. Every cancer was discovered by palpation. The average tumor size was 4.5 cm and 60–70% of axillae were node-positive. The axillary dissections were thorough and complete, often 25–30 nodes. The 5-year distant recurrence rate for node-positive patients was 50–70% and for node-negative 20–30%. There was no estrogen or progesterone receptor testing, no HER2, no tumor subtyping, no anything other than size and nodal status. There were a couple of relatively ineffective drugs available as chemotherapy. There was no DCIS, a disease that was just becoming a reality to a few pathologists.

I did radical mastectomies on every patient with breast cancer until 1974 when I was a surgical oncology fellow. At that point, due to pressure from the Women’s Movement (not a randomized trial), most surgeons switched to a muscle-sparing modified mastectomy.

In 1981, everything changed when Veronesi et al. published the Milan I Trial.^1^ At 5 years, survival was equal for Halsted mastectomy compared with segmental resection, axillary dissection, and whole breast radiation therapy (WBRT). The previous dogma, that you must remove the entire breast and more was about to change.

I was at the Van Nuys Breast Center at that time, and our group became early adopters of breast conservation based on that single paper. Breast conservation was one of the reasons for our success since most other surgeons in Los Angeles and elsewhere continued to do mastectomies. In 1985, the NSABP B-06 (National Surgical Adjuvant Breast Project), led by Bernard Fisher, confirmed that breast conservation was safe.^2^ Nevertheless, it still took another 10–15 years for breast conservation to be widely accepted in the United States.

By 1985, at the urging of our plastic surgery colleagues at Van Nuys who complained about the cosmetic results of our lumpectomies, a plastic surgical component was added to our initial resections. Our plastic surgeons lobbied that the appearance of the breast postoperatively was important. Initially, we fought them. Opening new planes and moving tissue into an area previously occupied by cancer was thought, at that time, to be non-oncologic and against all Halstedian training. We resisted at first but eventually embraced the change. We became oncoplastic surgeons.

We added the reduction excision when European visitors convinced us that they had been using it safely for a few years. From there, everything evolved at light speed.

By 2008, I had moved from Van Nuys to the University of Southern California and then to Hoag Memorial Hospital. During that journey, the reduction excision became routine and we evolved, conserving the breast for larger and larger tumors. Our philosophy became, if the breast and tumor could accommodate a reduction excision with a reasonable chance of a clear margin, we did it.

At first, we called it *radical conservation*, the opposite of radical mastectomy. But no one liked the use of the word *radical*. We defined radical conservation as breast conservation in a patient with a large, greater than 5 cm, often multifocal/multicentric tumor, that most surgeons would suggest be treated with mastectomy.^3^

In 2015, in the surgeon’s lounge at Hoag Memorial Hospital, we finally came up with a better term. We renamed the procedure *Extreme Oncoplasty*, which became the title of the 2*015 IS Ravdin Lecture* at the annual meeting of the American College of Surgeons.^4^ A few surgeons came up to me after that presentation and said, “I don’t like what you’re doing. It’s dangerous.” But we persisted.

## Present

Extreme oncoplasty is now a real thing. If you look on the internet, you will find thousands of articles, references, and discussions about it. In our paper, in this edition of the *Annals*, “Extreme Oncoplasty: Avoiding Mastectomy and Expanding the Limits of Breast Conservation—A Comparative Study of 718 Patients,” we used a prospective database to select every patient with breast cancer from 2008 to 2025 who had been treated with a reduction excision and who received WBRT. We divided them into two groups: those with tumors 5 cm and smaller (standard cases), and those with tumors greater than 5 cm (extreme cases). Distant recurrence, overall, and breast cancer survival were essentially the same in both groups. Local recurrence was slightly higher for the extreme cases, but it was not statistically significant.^5^ The size of the tumor did not make a difference in outcome.

In a previous paper, we compared patients with extreme tumors who had been treated either with reduction excision plus WBRT or with mastectomy without additional WBRT. Again, the results were similar between groups, as to local and distant recurrence and survival.^6^

What does this mean? It means that there should be far fewer mastectomies than we see now. Oncoplastic breast conservation can be done safely in most patients, even patients with large multiquadrant tumors and should be available across the country in all hospitals that treat breast cancer. Mastectomies should be reserved for patients who absolutely will accept nothing less, for patients who desire maximum risk reduction for genetic or other reasons, and for patients with tumors so extensive or breasts too small that a safe oncoplastic reduction cannot be designed, even with all the diagnostic and neoadjuvant tools currently available. Going forward, relatively few patients should truly require mastectomy.

## Future

The future brings many new tools, such as ctDNA. The concept of detecting breast cancer so early in the natural history of the disease, before it is visible on mammogram or MRI, means the possibility of systemic treatment before surgery and radiation therapy are ever necessary. Until then, we will continue to see new and improved early diagnostic tools. Artificial intelligence (AI) is already reshaping how we screen and diagnose. AI will not replace the clinician, but it will become an indispensable partner.

The future brings the possibility of in situ ablation using a variety of evolving tools. Surgical treatment will evolve and continue to de-escalate as systemic therapies become even more effective. Immunotherapy and novel systemic agents are already producing pathologic complete responses at rates unimaginable a generation ago. Cancer vaccines are in active development. These advances make it possible to envision a future where systemic treatment administered early enough means no surgery for a growing number of patients.

As breast cancer incidence among younger women continues to increase, the next generation of patients will look very different from the last. If current trends persist, driven by shifts in reproductive patterns, environmental exposures, and lifestyle factors, the demand for earlier detection, fertility-preserving strategies, and less disfiguring treatment will only grow. The tools we are building are not just innovations, they are necessities.

We are optimistic that the future will transform a diagnosis of breast cancer from a life-threatening event into a manageable condition, treated with non-deforming interventions that preserve quality of life. But we must be deliberate in ensuring equitable access to the best breast cancer treatments, not only in the United States, but worldwide. As we shift from survival to survivorship, quality of life must be a primary endpoint, not an afterthought.

We should save every breast we can and ensure that every woman, regardless of age, race, income, or geography has the opportunity to benefit from the future that we are building.
